# Application of mRNA Technology in Cancer Therapeutics

**DOI:** 10.3390/vaccines10081262

**Published:** 2022-08-05

**Authors:** Yesim Eralp

**Affiliations:** Research Institute of Senology, Acıbadem University, Istanbul 34457, Turkey; yeralp@yahoo.com

**Keywords:** mRNA vaccines, mRNA delivery platforms, cancer therapeutics, cancer immunotherapy, cancer specific immune response, clinical applications in cancer therapy

## Abstract

mRNA-based therapeutics pose as promising treatment strategies for cancer immunotherapy. Improvements in materials and technology of delivery systems have helped to overcome major obstacles in generating a sufficient immune response required to fight a specific type of cancer. Several in vivo models and early clinical studies have suggested that various mRNA treatment platforms can induce cancer-specific cytolytic activity, leading to numerous clinical trials to determine the optimal method of combinations and sequencing with already established agents in cancer treatment. Nevertheless, further research is required to optimize RNA stabilization, delivery platforms, and improve clinical efficacy by interacting with the tumor microenvironment to induce a long-term antitumor response. This review provides a comprehensive summary of the available evidence on the recent advances and efforts to overcome existing challenges of mRNA-based treatment strategies, and how these efforts play key roles in offering perceptive insights into future considerations for clinical application.

## 1. Introduction

During the past decade, major technological advances have enabled the use of mRNA-based therapeutics as a promising innovative approach in cancer therapy. Initial cancer vaccine trials date back to the 19th century with reports of tumor regression in patients injected with samples of erysipelas [1] and have continued since then with insufficient success. A dendritic cell-based vaccine directed against PSA, namely Sipuleucel-T, remains the only approved therapeutic cancer vaccine with limited clinical applicability [2].

Navigating the host immune system through induction of tumor-specific immunity by activating cytotoxic T cells to eliminate cancer cells is the mainstay of cancer immunotherapy. In order to induce an adaptive immune response, T cells have to recognize cancer-specific neoantigens processed by antigen presenting cells (APCs) such as dendritic cells or tissue macrophages, which delineate the trenches of immune-related cell killing. Thus, identification of tumor neoantigens has played a key role to establish the development of cancer vaccines [3,4].

mRNA vaccine is a recent innovative approach to cancer immunotherapy by encoding tumor-specific antigens to be introduced into APCs to synthesize the required antigens by the intracellular machinery. Early preclinical studies have shown generation of robust antitumor immune responses to mRNA-based cancer vaccines that are capable of cytotoxic activity [5,6]. Furthermore, mRNA vaccines are more advantageous in terms of production cost and feasibility as compared to DNA-based vaccines. They are less expensive, require less comprehensive and time-consuming manufacturing processes, and enable enhanced precision in tumor-directed genomic targeting as well as inability for DNA integration, which is a major drawback in alternative nucleic acid vaccines [7].

Despite the enthusiasm generated in the field of mRNA-based immune-oncology, many challenges remain to be addressed before this strategy can be adopted in routine practice. Numerous efforts are being placed to improve translational ability and stability through molecular engineering. Furthermore, much research is being carried out to optimize delivery systems in order to facilitate intracellular uptake and mitigate inherent immunogenicity ensued through degradation by extracellular ribonucleases [8]. In this review, data on current mRNA-based vaccines are discussed in the context of available evidence from preclinical and clinical studies, highlighting future prospects of incorporating this novel therapeutic strategy in cancer immunotherapy.

## 2. Cancer Immunology and Immunotherapy

The immune system comprises of elements of myeloid and lymphoid lineage such as lymphocytes and macrophages, which are specialized to generate an immune response against foreign-appearing structures in the host, including cancerous cells. When a tumor cell encounters the innate immune system, an inflammatory signaling cascade is initiated, which stimulates induction of dendritic cell maturation, immunostimulatory cytokine secretion, and natural killer cell activity. Once tumor cells are internalized by dendritic cells, these APCs interact with the microenvironment to present the neoantigen to cytotoxic T cells and B cells, which are subsequently activated to develop an antigen-specific immune response. The generation of an active adaptive immunity requires dendritic cell maturation and induction of danger signals from both apoptotic or necrotic cells and the TME during antigen processing [9,10]. Nevertheless, as tumors progress, the anticancer immunologic activity may be hampered by the upregulation of various checkpoints and immune-suppressive elements of the host immune system, which are naturally programmed to balance any excessive immune activity against the host. Recently identified inhibitory immunoreceptors, such as lymphocyte-activation gene 3 (LAG-3) and T cell immunoreceptor with immunoglobulin and ITIM domain (TIGIT), indoleamine 2,3-dioxygenase (IDO) controls immune activity by suppressing tumor-specific T and B lymphocytes, resulting in a shift towards generation of an immune-suppressive stroma comprising of exhausted T cells, which lack the ability to generate an anti-tumor immune response; as well as myeloid and lymphoid elements of the immune system associated with immune escape, namely regulatory T cells (Treg), immature dendritic cells, M2 macrophages, and myeloid derived suppressor cells (MDSC) [11]. It has been shown that cancer cells tap into the host immune system to overcome immune-mediated cell killing by switching the tumor microenvironment to an immunosuppressive or immune-cold phenotype mediated by several cytokines and molecules [11,12,13].

Immunotherapy refers to all types of treatment strategies aiming to restore immune dysregulation or to modulate the host immune system to destroy cancer cells. The current immunotherapy approaches involve strategies that aim to release the brakes on the host immune regulatory systems by inhibiting checkpoint upregulation by programmed cell death protein-1 (PD-1), programmed cell death ligand-1 (PD-L1), or cytotoxic T lymphocyte antigen-4 (CTLA) inhibitors, or to stimulate the immune system to generate a cancer-specific response by cancer vaccines or to administer ex vivo activated autologous or allogeneic immune cells that target cancer cells, such as chimeric antigen receptor-T (CAR-T) cells or engineered natural killer (NK) cells, otherwise referred to as adoptive cell therapy [14,15,16,17,18,19].

## 3. The Evolving Role of mRNA Technology in Cancer Immunotherapy

Nucleic acids in the form of RNA or DNA, whether exogenous from bacterial or viral causes, or endogenous, shed from cancer cells are capable of inducing variable degrees of immune response [20]. Cancer antigens, whether as whole-cell lysates, peptides, or as nucleic acids intended to translate into the structural protein of the antigen itself, can be delivered into the host in order to generate a cancer-specific immune response, otherwise referred to as a “cancer vaccine”. In contrast to peptide-based or whole cell vaccines, nucleic acid vaccines are more advantageous since they enable delivery of multiple or full-length tumor antigens, leading to a broader immune response.

In vitro transcribed RNAs, which are proven to have a wide applicability in SARS-CoV-2 vaccinations, have recently gained interest as cancer vaccines due to their versatility to encode chimeric peptide structures, allowing for targeting cancer cells with diverse and complex mutational structures. Furthermore, mRNA vaccines have emerged as an appealing alternative to DNA vaccines not only due to their ability to be translated in both dividing and nondividing cells, but also due to their safety since they cannot integrate into the host genome [21].

**Figure 1:**
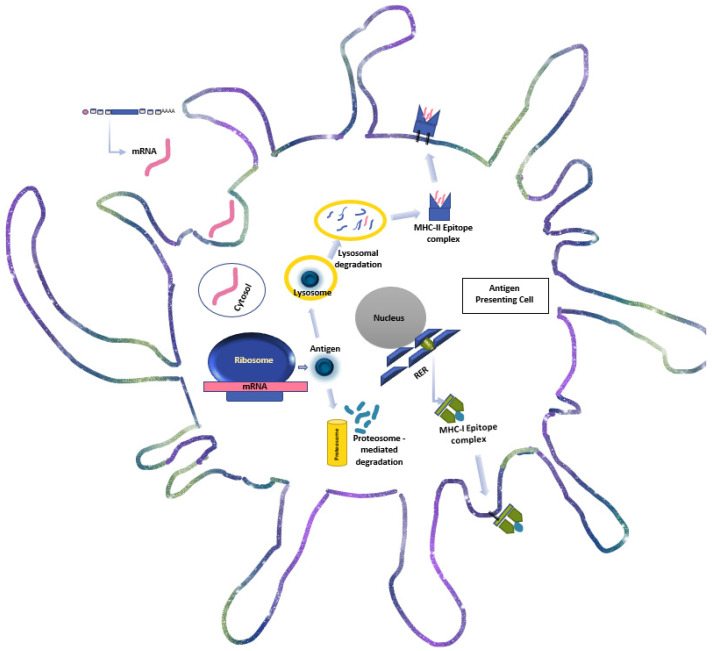
Schematic diagram of intracellular events in mRNA processing by the antigen presenting cells. mRNA enters the cell through the cytosol and translated by the ribosome into the encoded antigen. The antigen is then: (A) degraded into small protein fragments and epitope by the proteosome, which combine with the MHC-I complex at the rough endoplasmic reticulum and traffics to the cell membrane for presentation to naive CD8 (+) cytotoxic T cells; (B) either exocytosed to re-enter the APC through endocytosis or enters the autophagic pathway. Then, the antigen is split into fragments and its epitopes by lysosomal degradation. These epitopes bind with the MHC-II complex and are transferred to the cell membrane to activate naive CD4 (+) T lymphocytes. (MHC: major histocompatibility complex, RER: rough endoplasmic reticulum).

As synthetic mRNA enters the host cells through the cell membrane or by endocytosis, translation to the peptide of interest occurs within the cytosol. This protein structure, which is undistinguishable from the product of endogenous mRNA, undergoes post-transcriptional modifications eventually leading to degradation by intracellular compartments. These peptides are then presented on major histocompatibility complexes (MHC) of the antigen-presenting cells to be introduced to the effector cells of the host immune system to induce cancer-specific killer T cells along with activated helper T lymphocytes and NK cells [22] (Figure 1). In addition to the generation of a cancer-specific immune response, exogenous mRNA helps to maintain an immune-friendly tumor microenvironment (TME) by triggering secretion of type I Interferon (IFN) and other inflammatory cytokines through activation of toll-like receptors (TLR) and retinoic acid-inducible gene I (RIG-I) [23]. Furthermore, mRNA constructs can be engineered to express proinflammatory cytokines including, but not limited to Interleukin 2 (IL-2) IL-7, IL-12 and IL-15, which act synergistically to enhance generation of antigen-specific CD 8 + cytotoxic T cells, increase the ratio of active CD8 cells to immune suppressive Tregs, and induce memory T cells for a long-lasting immune response [24,25,26,27,28]. In addition, mRNAs are also being developed to encode monoclonal antibodies (mAbs), which have an established role as a passive targeted immunotherapy approach for various cancer types such as Her-2 positive breast cancer and lymphomas. There is in vivo evidence that suggests mRNA encoded mAbs are indeed able to induce a more sustained antitumor effect as compared to their recombinant equivalents in murine models [29,30]. These constructs can be modified to encode bispecific mAbs comprising an anti-CD3 Fv and a tumor-specific Fv, which are able to redirect T cells to the TME to elicit a stronger immune-mediated tumor cell killing [31].

## 4. The Immunogenicity and Molecular Biology of mRNA-Based Immunotherapy

Therapeutic mRNAs are produced through in vitro transcription (IVT) catalyzed by DNA-dependent RNA polymerase, which selectively recognize the promoter region of DNA templates [32]. The end product is a naked mRNA strand, which should be modified to optimize the stability and translational ability. These modifications include capping the 5′ end, optimizing the sequence of the untranslated translating regions, and adding a poly-A tail [33]. Nevertheless, these alterations and byproducts generated during the IVT process may impede the desired antitumor response through activation of the innate immune system, leading to the recognition of the modified mRNA molecules as nonself, as well as interference with the transcriptional capacity by cellular stress mechanisms [34].

As the first line of defense against external and internal pathogens, the innate immune system initiates a cascade of events subsequently triggering adaptive cancer-specific immunity. Endogenous and exogenous non-self-nucleic acids are recognized by intracellular pattern recognition receptor family (PRR) comprising TLRs, RIG-I-like receptors, nucleotide-binding and oligomerization domain (NOD)-like receptors, C-typelectin receptors, absent-in-melanoma 2 (AIM2)-like receptors, and the cyclic GMP-AMP synthase. Activation of PRRs localized in the cytosol and endosomal compartment in turn lead to transcriptional activity of several proinflammatory cytokines and chemokines, and stimulation of transcription-independent intracellular pathways such as autophagy, apoptosis, and phagocytosis. Studies with synthetic nucleic acids to manipulate the immune system have shown that different sequences of dsRNA (siRNA) varying in length induce distinct immune responses, which may be in opposite directions [20,35,36]. Therefore, purity and nucleotide composition of therapeutic mRNAs play a significant role in generating an optimal immune response.

### 4.1. mRNA Vaccine Structure

**Figure 2:**
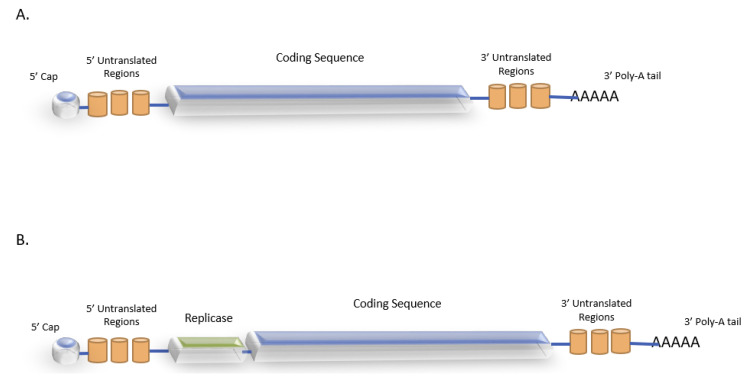
Schematic structure of an mRNA vaccine construct: (**A**) non-replicating and (**B**) self-replicating, 5′ cap—all eukaryotic mRNA has a cap that contains an m7GpppN structure, preserved throughout evolution. The cap structure not only prevents degradation, but also assists in binding with the eIF to activate translation. Untranslated regions regulate the translational efficiency, whereas the coding sequence contains codons that encode the gene of interest. The poly-A tail acts to maintain the stability of the RNA molecule [37,38,39,40].

Two types of mRNA-based vaccines are available: nonreplicating (NRM) and self-replicating mRNA (SRM) vaccines, which are composed of a universal 5′ cap, 3′ and 5′ noncoding regions, an open reading frame, and a 3′ poly-A tail. While the cap structure protects the mRNA from quick degradation and induces IFN-mediated immune responses, the untranslated regions regulate the translational efficiency of mRNA. The poly A tail plays a significant role in the translation by regulating the stability of mRNA. Enrichment of G:C content and utilizing modified codons in the ORF constructs and optimizing the length of the poly-A sequence are some of the structural modifications that promote a translational process [37,38,39,40] (Figure 2). The NRM, though technically less demanding to produce, has the disadvantage of limited activity and stability, which can be overcome to a certain extent by structural optimization. SRM vaccines differ from NRMs by including an extra construct that encodes a replicase component. Generally, these vaccines are produced through engineering of single-strand RNA viral structural genes, which have been substituted by the gene of interest (i.e., cancer antigen), while keeping the nonstructural genes (i.e., replicase), leading to a high level of antigen expression within a delivery system. Picornaviruses, alphaviruses, and flaviviruses are the most common RNA viral systems employed to generate SRM vaccines [5,41,42,43,44].

### 4.2. mRNA Delivery Platforms

Although mRNA technology is a promising tool for cancer immunotherapy, a number of challenges have to be faced to facilitate an effective immune response. First, the large and negatively charged RNA molecule has to cross the cell membrane, which is a significant barrier to intracellular delivery due to its negative charge. Once mRNA enters the cell, there is a high risk for degradation through ribonucleases, which are abundant throughout the skin and systemic circulation. Although delivery of naked mRNA is possible through intradermal, subcutaneous, and intramuscular routes, the efficacy of such approaches is hindered by a short half-life, rapid degradation and inadequate immune response due to ineffective access to intracellular compartments. Therefore, an efficient delivery is crucial to achieve favorable therapeutic potential. Therapeutic advances in mRNA technology have been linked to the development of various nanotechnological delivery systems that have been engineered to ensure optimal translational capability [5,21,45].

#### 4.2.1. Synthetic Systems

1. Lipid-based Delivery Systems

Lipid-based materials are the most extensively investigated delivery systems for RNA-based therapeutics. Referred to as lipid nanoparticles (LNP), these structures consist of a cationic or more recently a pH-dependent ionizable lipid layer; a polyethylene glycol (PEG) component; phospholipids and cholesterol [45,46]. The ionizable amino lipid layer is designed to obtain a positive charge as pH drops, facilitating endosomal uptake of the liposome, while retaining encapsulation of the negatively charged mRNA molecule. The PEG molecule plays a significant role in preventing macrophage-mediated degradation, together with providing stability along with cholesterol [47,48,49]. The structure of the amino lipid component plays a key role in delivery efficacy, tolerance, and tissue clearance [50]. Efforts to optimize LNP delivery of mRNA vaccines have yielded efficient RNA delivery in cell lines, and strong, long-lasting humoral immune responses against several viral pathogens in murine models [51,52,53]. Clinical studies with two LNP-based mRNA vaccines against the SARS-CoV2 virus during the pandemic have confirmed the favorable results, showing a strong efficacy across different populations, leading to regulatory approval despite the short-term follow-up period [54,55].

Nevertheless, as part of a cancer vaccine, LNP design should further be developed to deliver the mRNA cargo specifically to antigen-presenting cells, while preventing degradation and retain effective translational capacity. Moreover, the amino lipid structure should be biodegradable to prevent toxicity and allow for multiple dosing at the same time. Emerging evidence from preclinical studies suggest that LNP mRNA vaccines provide robust antigen-specific antitumor with memory T cell responses by specifically targeting dendritic cells, leading to prevention of tumor growth in murine models [56,57,58,59].

2. Polymer-based Delivery Systems

Polymeric materials and dendrimers, modified with nanotechnologic fatty side chains to reduce toxicity and avoid enzymatic degradation in vivo, have gained popularity to deliver mRNA as vaccines against fatal viral pathogens such as HIV, Zika, Ebola, and H1N1 Influenza [60,61,62,63,64]. Polymeric structures surrounded by a PEG outer shell have been used in murine models to deliver an antiangiogenic RNA sequence, which was shown to inhibit growth in a pancreatic cancer model [65]. Similarly designed mRNA vaccines have been shown to effectively translate into tumor-associated antigens in vivo [66]. Furthermore, a polymer-based RNA vaccine encoding PTEN has successfully been introduced into several castration-resistant prostate cancer models and has been shown to inhibit tumor growth by restoring PTEN function [67].

3. Peptide-based Delivery

Cell-penetrating peptides (CPPs) are cationic peptides that can translocate through the cell membrane independent of receptors and can transport proteins, small organic molecules, nanoparticles, and oligonucleotides. Because of a favorable safety profile and efficient transfection capability, CPPs represent a promising class of nonviral delivery vectors [68,69,70]. Nevertheless, low cell and tissue selectivity, and impaired internalization of the cargo by conjugation through different cellular layers limit efficient clinical application [71]. Recent efforts have been focused on identifying the most optimal CPP for enhanced immune activity. Protamine is a cationic peptide that can prevent lysosomal degradation during delivery of RNA. Protamine-based deliveries have been shown to induce a strong immune response through toll-like receptor 7 activation [72,73]. More recently, advances in biotechnology have led to promising developments in peptide-based mRNA delivery. For example, a pegylated cationic KL4 peptide complex in powder form has been successfully used as an aerosolized delivery system for pulmonary delivery [74]. Furthermore, an optimized GALA-peptide conjugated mRNA encoding the Ova peptide exhibited a strong APC uptake and an efficient endosomal escape, leading to enhanced antigen-specific T cell activity and dendritic cell maturation compared to naked RNA or different peptide complexes [75].

#### 4.2.2. Biological Systems

1. Ex Vivo Transfected Cellular Systems

Immunotherapy against cancer requires transfection of APC with specific antigens or nucleic acids such as mRNA, which translate into tumor-specific antigens. Although in vivo transfection via the intramuscular, intravenous, or subcutaneous routes are possible, the immune response generated is usually weak and unsustained. Therefore, ex vivo transfected engineered dendritic cells or chimeric antigen receptor T cells have been developed as cancer vaccines or adoptive cell therapy strategies to target cancer cells once introduced in the host [76].

- Dendritic Cells

Dendritic cells play a crucial role in reprogramming the immune system by their ability to uptake and present the tumor antigens, leading to generation of potent effector cell activity directed against cancer cells. Additionally, mature dendritic cells are capable of modulating chemokine- and cytokine-induced lymphoid activation, which are strictly relevant for a systemic and sustainable anticancer immune response. As autologous cancer vaccines, dendritic cells are harvested from the host by apheresis, isolated from mononuclear cells or progenitor stem cells, subsequently stimulated by various cytokines to achieve maturity, followed by transfection with specific antigens as nucleic acids or peptides. Numerous efforts have been focused on methods to achieve a stronger immune response through more efficient antigen presentation, migration to required lymphatic tissues, and induction of a stronger cytokine production through generation of Notch differentiated dendritic cells with engineered receptor expression capability using clustered regularly interspaced short palindromic repeats (CRISP-R) gene editing and RNA interference, as well as the use of optimized maturation cocktails [77,78,79]. Ex vivo transfection of mRNA-loaded dendritic cell vaccines against a variety of tumor specific antigens such as telomerase reverse transcriptase (TERT) and the melanoma cell line B16F10 have led to generation of a strong antitumor immune response in murine melanoma models [80,81].

b. CAR-T Cells

Chimeric antigen receptor (CAR)-modified T cells represent a novel adoptive cell therapy approach that has been shown to effectively target tumor cells leading to a potent immune-mediated cancer cell killing [82]. CAR-Ts confer several advantages over natural host immunity by MHC independent tumor antigen presentation, more potent cell receptor binding, and ability to bypass escape mechanisms such as HLA downregulation [83]. Direct transfection by electroporation or viral systems has been utilized to deliver CAR-encoding mRNA to generate cancer-specific CAR-T cells [84]. More recently, RNA optimization by nanoparticles and gene editing through CRISP-R technology has been utilized to engineer CAR-Ts that have improved stability and transfection ability [85,86]. Preclinical studies investigating ex vivo transfection of patient-derived T cells by retroviral constructs to deliver mRNA encoding bi-CARs targeting tumor-specific epitopes have shown that the engineered CAR-Ts are capable of recognizing target antigens and overcoming escape variants, eventually leading to improved survival in a glioblastoma (GBM) murine model [87]. Furthermore, profound cytotoxic cell lysis has been demonstrated with RNA transfected CAR-T constructs expressing CD19 in xenograft models with leukemia [88,89], extensively reviewed elsewhere by Rajan et al. [90].

2. Viral Constructs

Viral constructs generated from RNA viruses have been evaluated extensively as self-replicating RNA (SRM) vaccines against several cancer types. Single-strand RNA viruses including alphaviruses, flaviviruses, and rhabdoviruses can be engineered to form naked RNA replicons and recombinant viral-like particles (VLP), which are capable of producing a high level of tumor antigen expression in APCs, in turn leading to a strong immune response [91,92]. An SRM vaccine comprises a replicon carrying the gene of interest in conjunction with the replicase gene and a defective virus encoding structural genes, forming VLP in a packaging cell construct. The VLP, taken up by APCs when introduced into the host, deliver self-replicating RNA constructs to the cytosol by receptor-mediated endocytosis, leading to a high level of RNA production and tumor–antigen expression through translation [44,93,94]. Preclinical studies evaluating the role of replicon-based SRM vaccines have shown the success in eliciting strong humoral and cellular immune responses against several cancer types in xenograft models harboring Her-2 neu breast cancer, prostate cancer, GBM, and human papilloma virus (HPV)-induced tumors [95,96,97,98].

## 5. Clinical Applications

### 5.1. Personalized mRNA Vaccines

#### 5.1.1. Naked mRNA Vaccines

Direct intradermal injection of naked mRNA sequences was shown to effectively produce the encoded protein leading to generation of tumor-specific functional immune response in various cancer types including lung cancer and prostate cancer, hence were introduced as an alternative vaccination method [99,100]. Early clinical studies of naked mRNA fortified with protamine stabilization showed a favorable immune response in 50% of the cohort comprising seven patients with melanoma, and a complete clinical response in one patient [101]. Similarly, another multiplex mRNA vaccine encoding carcinoembryonic antigen (CEA), mucin 1 (MUC1), human epidermal growth factor receptor 2 (Her-2), melanoma-associated antigen (MAGE), survivin and telomerase as tumor-associated antigens was evaluated in 30 patients with advanced renal cell carcinoma (RCC). Vaccinations were reported to be feasible with specific CD4 and CD8 immune responses and no serious toxicity. Median survival was 29 months, with approximately one-third of the cohort alive at 4 years [102]. Long-term follow up extending to 10 years revealed a strong correlation of immune responses with prolonged survival [103]. A sequence-optimized RNA vaccine encoding five non-small cell-associated tumor antigens, BI1361849 was investigated as part of a combination strategy with radiotherapy in stage 4 lung cancer patients responding to platin-based chemotherapy. Immunologic analysis revealed 40% of patients reaching the prespecified threshold of two-fold increase in functional tumor-specific CD4 and CD8 cell generation. The best overall response rate (ORR) was disease stabilization in 46% of patients [104].

#### 5.1.2. LNP mRNA

The only available clinical data have been reported by Sahin et al. [105], who have investigated the efficacy of a liposomal RNA vaccine (FixVac), with and without PD-1 inhibition in the dose-escalation phase I Lipo-MERIT trial. FixVac, which is specifically designed to target dendritic cells, encodes four tumor antigens associated with malignant melanoma. All patients enrolled in this trial had previously received immune checkpoint inhibitors. In the interim analysis, 16% objective responses were observed in the monotherapy arm of 25 patients. The vaccine showed synergistic activity with PD-1 inhibition showing an ORR of 35%, which increased to 50% with increasing dose of the FixVac. Translational work on some patients with long-term immune monitoring has indicated generation of memory T cells, along with helper and cytotoxic T cell response in some responders. Data from this trial and others are awaited to provide further insight on the clinical applications of LNP mRNA vaccines and optimal combinations.

#### 5.1.3. Dendritic Cell-Based Vaccines

Early clinical trials with RNA transfected dendritic cells have shown that vaccination with this strategy is feasible and is able to stimulate tumor-specific T cell responses in vivo. Based on encouraging preclinical data, a phase Ib study evaluated the role of PSA encoding mRNA dendritic cell vaccine in 16 patients with metastatic castration-resistant prostate cancer patients [106]. There was a significant increase in CTL response seen in all patients after completion of therapy and an ongoing prostate-specific antigen (PSA) response in six of seven evaluable patients who did not have subsequent therapies. An autologous RNA transfected DC vaccine was investigated in a pioneering trial in 10 patients with metastatic RCC. Confirmatory to the previous trial, there were strong antitumor T cell responses against three RCC antigens generated in five out of six patients who were evaluable [107]. Carcinoembryonic antigen is a frequently expressed tumor antigen in gastrointestinal tumors. In a phase Ib/II trial, 24 patients with resected hepatic metastases of colorectal carcinoma were vaccinated with CEA-encoding DC. In addition to the immune responses, there was one complete response and two minor responses with a clinical benefit ratio of 25%. The median RFS in the phase II cohort was reported as 122 days [108]. A different approach from the same group evaluated CEA transfected mRNA vaccination in three patients with localized pancreatic carcinoma, who were operated after neoadjuvant chemoradiation. All but one patient received the planned 6-month treatment, and no side effects were noted. Patients were reported as disease-free at approximately 4 years from diagnosis [109]. A DC-based mRNA vaccine encoding CD40Ligand, CD70, and TLR4 (TriMixDC), and transfected with melanoma associated genes (MAGE, Tyrosinase, gp100-TriMix-MEL) was evaluated in a cohort of advanced melanoma patients. There were two partial responses (13.3%), and the median progression-free survival (PFS) and overall survival (OS) were reported as 5 months and 14 months, respectively [110]. Based on the encouraging early data, the vaccine was used in an earlier disease setting following resection for stage III/IV melanoma. In the investigational arm, 21 patients received four vaccinations with a booster over 6 months, whereas the control group had no adjuvant therapy. Although vaccination was deemed to be feasible, the study was closed early due to futility. In evaluable patients, the median time to progression was 8 months, with more early relapses in the vaccine group and 13 months in the control group. Nevertheless, the 1-year disease-free survival (DFS) appeared to be higher in the vaccine arm: 71% vs. 35%, respectively [111]. TriMix-MEL was also evaluated as part of a combination with ipilimumab in pretreated patients with melanoma who had not received prior immune checkpoint blockade. Out of 39 patients, there were 8 patients with a complete response (CR) and an ORR of 38%. Reaching the primary endpoint of 6-month DCR, the median PFS and OS were 27 and 58 weeks, respectively [112]. A personalized mRNA transfected DC vaccine used in combination with PD-1 inhibition and low dose cyclophosphamide also confirmed generation of a high level of tumor-specific immune response and favorable survival outcomes in a cohort comprising 10 patients with lung cancer and GBM [113]. Both trials provide strong insight into the combined use of vaccines with immune checkpoint blockade, which deserve further investigation.

#### 5.1.4. Viral-Based Self-Replicating mRNA Vaccines

Self-replicating viral-based constructs from RNA delivery have been shown to be safe and feasible in early clinical trials for both infectious diseases and cancer immunotherapy [114]. Early clinical studies with viral constructs have utilized alphavirus-based viral replicon particles (VRP) more frequently, which were designed to efficiently express the desired tumor antigen in high amounts following targeted uptake in DCs. In fact, a first- in-human study with a replication incompetent Semliki Forest replicon encoding HPV-associated antigens E6 and E7 showed strong antigen-specific interferon-gamma responses in three cervical in situ neoplasia patients [115]. Furthermore, one of the initial studies evaluated the feasibility of a CEA-encoding alphavirus particle in 30 patients with advanced solid tumors who received four doses with 3 weekly intervals. As a main endpoint, investigators were able to deduce that multiple injections of the VPR-CEA (Tricom-CEA) were feasible and could generate specific CD8 and CD4 T cell responses. However, the level of immune responses remained stable during the booster period in some patients who were able to receive them, suggesting that more than four injections would probably be unnecessary. Yet, there was one responder in the cohort and two patients with stabilization of disease in this heterogeneous cohort with some correlation between long-term efficacy and the level of immune response [116]. Long-term follow up results of a separate cohort comprising stage III colorectal cancer patients treated with the same vaccine were recently reported. In parallel with the phase I–II data, a specific immune response was induced in all patients, which was higher compared to stage IV patients from the previous trial. After a median follow up of 5 years, all patients were reported to be alive, with a 25% recurrence rate [117]. The same viral construct was used to produce a VPR encoding the transmembrane and extracellular domains of the Her-2 receptor, which was evaluated in a phase IB-II trial including patients with advanced Her-2 (+) breast cancer. All patients had received prior her-2 blockade, and one of the two cohorts included in the trial received combined anti-Her2 therapy with the vaccine, while cohort one received vaccine as monotherapy, given as three injections every 2 weeks. Investigators reported detectable levels of anti-Her2 immune responses, which unfortunately did not translate into relevant clinical responses, with median PFS of 1.8 and 3.6 months in cohorts 1 and 2, respectively [118]. Limited data from small studies including prostate, lung, and colorectal cancer suggest that VPR-based mRNA vaccines are able to generate antigen-specific immune responses, the level of which may be correlated with the outcome [119]. Still, this area of mRNA technology requires further investigation before finding a place in the immunotherapeutic landscape for cancer patients.

### 5.2. mRNA-Engineered Cellular-Based Immunotherapy and Gene Editing

Engineered CAR-T cells have revolutionized adoptive cellular therapy in hematologic cancers. The application of this technology to other immune cells has led to the development of viral transfected CAR-Ms (CAR-macrophages) that could potentially be used against solid tumors. Despite disadvantages in the manufacturing process, in vitro transcribed, mRNA-based CAR-encoding immune cells represent a safe and effective alternative to the first- and second-generation CAR-T cells that are currently approved for clinical use. Lipid nanoparticle delivery systems, as described previously, have been successfully utilized to deliver anti-CD19 coding mRNA in M1 macrophages and cytotoxic T cells [86]. RNA CAR-T cells have the advantages of rapid production and multiple administrations leading to enhanced efficacy, which has overcome several limitations pertaining to the routine use of CAR-T cells in the clinic. Furthermore, advances in genome editing have also refined selective targeting by immune cells. The CRISP/Cas9 system is a novel editing tool that can be utilized to select and delete the desired genome sites or result in knockdown of several genes through epigenetic silencing in order to enhance CAR-T efficacy [120,121]. Nevertheless, early phase I trials in various solid tumors comprising pancreatic and breast cancers have yielded unsatisfactory outcomes with transient responses despite generation of immune activity [84].

A list of ongoing trials on different RNA-based therapeutics is provided in Table 1.

## 6. Boosting Immune Response

### 6.1. Modulation of the Tumor Microenvironment

The tumor microenvironment (TME) plays a major role in controlling the cancer-immunity cycle through interaction with the signaling pathways leading to generation of a tumor-specific immune response. In some circumstances, mRNA can act as a tumor promoter, whereas, in other instances it can be modified to help generate an immune-friendly environment. In fact, tumor-derived mRNA has been implicated in activating angiogenesis under hypoxic conditions to promote tumor growth [122]. Furthermore, mRNA modification by N6-methyladenosine (m6-A), a redundant modification of eucaryotic mRNA, has been shown to be involved in generation of stemness property of cancer cells, promoting tumor growth and resistance to immunotherapy. Accumulating data suggests that targeting distinct m6-A regulators may reprogram the TME through secretion of immune-activating cytokines, upregulating costimulatory receptors and skewing the immune cell population toward an activated state by increasing the ratio of mature dendritic cells, M1 macrophages and Th1 cells to Tregs and MDSC [123]. In addition, distinct formulations with encoding mRNA have been shown to enhance immune-mediated cell killing through TME modulation. Research on the immunomodulatory use of RNA has been mainly focused on intratumoral or systemic delivery of mRNA engineered to produce costimulatory cytokines and receptors. Interleukin-2, an inflammatory cytokine playing a key role in differentiation and generation of effector T cell responses, has been used for the treatment of various tumor types since the turn of the century. Nevertheless, the unfavorable response–toxicity ratio with potentially fatal side effects has hampered its widespread use in routine practice and led to the adoption of alternative treatment strategies. Preclinical studies focusing on engineered mRNA encoding IL-2 in conjunction with other immune stimulatory cytokines such as IL-7, IL-15, and IL-12 used alone or as part of a combined approach with cancer vaccines, and adoptive T cell infusions, have been shown to generate synergistic immune activity in murine models with the advantage of an improved toxicity profile [24,26,124,125]. mRNA can also be used to transfect immune cells with costimulatory ligands including CD-40L and CD-70, enhancing immune-mediated cellular cytotoxicity in experimental models bearing several cancer types [126,127,128]. Advances in technology have also led to the production of fusion constructs, which have the ability of targeting tumor cells and the microenvironment simultaneously. Engineered mRNA encoding bispecific monoclonal antibodies expressing antitumor proteins, with receptors targeting effector T cells and immune checkpoints such as PD-1 and CTLA-4, and dendritic cells transfected with mRNA encoding agonistic ligands such as glucorticoid-induced TNFR-related protein (GITR), have been shown to elicit strong and sustained immune responses in experimental models [31,129,130]. Data from early clinical trials evaluating these novel strategies are awaited with enthusiasm.

### 6.2. Potential Combinations

Though mRNA fusion transcripts have provided an unprecedented opportunity to target multiple pathways in the cancer-immunity network, further combination strategies are required to ensure enhanced cancer cell killing. Energetic efforts are being placed to target several inhibitory elements of the TME, comprising angiogenesis, immune checkpoints, desmoplastic reaction, and fibrosis ensued by focal adhesion kinase (FAK) phosphorylation, epithelial–mesenchymal transition, and inhibitory signal transduction through phosphatase and tensin homolog (PTEN) loss or Myc activation [11,131]. There are numerous early-phase clinical trials evaluating the feasibility of combination approaches utilizing cancer vaccines and adoptive cell treatment with checkpoint inhibitors and antiangiogenic agents. Cytotoxic chemotherapy at various dose levels is an integral part in many of these studies. In fact, an ongoing clinical trial (NCT04503278) with an autologous CAR-T combined with an RNA-based cancer vaccine targeting claudin 6, has yielded encouraging activity in patients with refractory testicular or ovarian cancer [132]. Despite the theoretical advantage, an optimal combination has yet to be proven, rendering this as an active area of ongoing research. A comprehensive overview of ongoing and completed clinical trials has been provided elsewhere [133].

## 7. Conclusions and Future Perspectives

The advent of nanoparticle technology and genome editing tools has led to the generation of novel methods for RNA-based treatment. RNA can not only be modified to be used as a drug in itself, but can also serve as an efficient platform to deliver genomic information. During the last two decades, energetic efforts have been placed to optimize methods of mRNA-based gene therapies. Advances in nanotechnology have led to developments in delivery platforms, yielding encouraging results in preclinical and clinical studies for the treatment of a wide spectrum of diseases, including viral to bacterial pathogens, together with rare conditions related to genetic disorders and cancer.

Nevertheless, instability, impaired translational capacity, and lack of effective delivery methods have remained as major challenges, which require further research before this approach gains wide clinical applicability. The success of mRNA vaccines against SARS-CoV-2 during the recent pandemic has generated renewed enthusiasm to exploit this technology further. Despite the evident potential in the prevention of infectious diseases, the evolution of mRNA-based therapeutic strategies in oncologic care have lagged behind antiviral indications due to inadequate clinical responses. However, IVT RNAs delivered in various platforms as cancer vaccines or adoptive cell therapies have proved to be attractive and versatile tools to elicit cancer-specific immune responses, not only through effective antigen presentation, but also induction of an immune-friendly TME. Furthermore, there is early evidence suggesting that the clinical efficacy of mRNA-based systems can be enhanced through novel combinations with different anticancer strategies, including immune checkpoint inhibitors and chemotherapy.

Looking beyond cancer vaccines, advances in mRNA technology and gene editing have unraveled distinct innovative strategies for future development across several cancer types. Accumulating data from well-designed preclinical studies suggest that exogenously produced short, noncoding RNA fragments comprising antisense oligonucleotides (ASO), short interfering RNA (siRNA), and microRNA (miRNA) can be modified to target mRNAs for a wide array of genomic functions ranging from epigenetic silencing to restoration of inactive tumor suppressant genes such as TP53 or PTEN [134,135]. Another exciting advance in gene therapies with LNP-based mRNA platforms edited for aberrant genome targeting or protein replacement therapy has been the modification for specific organ targeting, a process called SORT for diverse cellular origins [136].

The oncology community is eagerly awaiting validated novel mRNA-based combinations for enhanced anticancer activity. The versatility of mRNA platforms and rapid production capacity of clinical grade products underscores the potential role of various mRNA therapeutic approaches in the future of cancer treatment.

## Figures and Tables

**Table 1:** Ongoing trials with mRNA-based therapeutics.

Trial ID	Trial Design	Target Patient Population (*n*)	Cancer Type	Investigational Treatment	Primary Outcomes	Trial Responsible Party/Collaborators
**Cancer vaccines:**						
NCT05192460	Phase I; Dose escalation and expansion with mRNA vaccine (PGV002)	Adult patients (*n*: 36)	Advanced gastric cancer, esophageal cancer, and liver cancer	Dose expansion: vaccine + PD-1/L1 inhibitor	Safety Tolerability Feasibility	Affiliated Hospital of the Chinese Academy of Military Medical Sciences, China
NCT05202561	Phase I; Open label; 2 arms; Arm I: mRNA cancer vaccine	Adult patients with HLA-A11:01 or C08:02 subtype (*n*: 10)	Refractory advanced solid tumors with KRAS mut	Arm II: vaccine + PD-1 inhibitor (Navuilumab)	Safety Tolerability Feasibility	Bengbu Medical College, China
NCT04534205 AHEAD-MERIT	Phase II; Open label nonrandomized 2 arm with run-in dose evaluation; mRNA vaccine + PD-1 inh vs. PD-1 inh monotherapy	Adult patients (*n*: 285)	Unresectable recurrent or metastatic HPV16+ HNSCC expressing PD-L1 with CPS ≥ 1	BNT113 (HPV 16 B6/7 mRNA vaccine) + Pembrolizumab	Run-in: Safety Phase II: OS and ORR; QoL	BionTech SE
NCT03313778 KEYNOTE 603	Phase I, Open label, dose escalation mRNA-4157 vaccine monotherapy (Part A); combined with PD-1 inhibitor (Part B, C, D);	Adult patients (*n*: 142)	Part A: clinically disease-free after early cancer diagnosis Part B, C: unresectable (locally advanced or metastatic) solid malignancies NSCLC, SCLC, HPV (-) HNSCC; Bladder urothelial; melanoma; MSI-H; high TMB Part D: resected melanoma	Part B, C, D: mRNA-4157 vaccine (lipid encapsulated mRNA vaccine encoding 20 tumor neoantigens) + Pembrolizumab	Safety	Moderna TX, Inc.
NCT01686334 WIDEA	Phase II randomized; Open label mRNA dendritic vaccine vs. surveillance	Adult patients (*n*: 130)	AML with minimal residual disease following front-line chemotherapy (morphological CR or CRi)	Autologous dendritic cells loaded by mRNA electroporation with the Wilms’ tumor antigen (WT1)	OS	Antwerp University Hospital; Belgium
NCT04526899	Phase II randomized; Open label BNT111 and Cemiplimab Combination vs. single agents	Adult patients (*n*: 180)	Anti-PD-1-refractory/Relapsed, Unresectable Stage III or IV Melanoma; ≥1–5 prior lines treatment including nivolumab/pembrolizumab or BRAFinh	BNT111 and Cemiplimab Combination vs. BNT111 (mRNA vaccine encoding 4 melanoma tumor antigents- NY-ESO-1, MAGE-A3, tyrosinase, and TPTE) vs. Cemiplimab	ORR	BionTech SE
NCT04573140 PNOC020	Phase I; dose escalation; Autologous LP-mRNA tumor vaccine	Pediatric and adult Patients (*n*: 28)	Newly diagnosed adult MGMT unmethylated glioblastoma and Pediatric High-Grade Gliomas (pHGG); <3 cm residual tumor following surgery and completed chemoradiation	Autologous total tumor mRNA and pp65 lysosomal associated membrane protein (LAMP) loaded lipid particles (liposomal vaccine)	Feasibility, Safety, Dose finding	University of Florida
NCT04911621 ADDICT-PedGLIO	Phase I–II mRNA loaded autologous mRNA dendritic cell vaccine	Pediatric Patients (Aged ≥ 12 months and < 18 years) (*n*: 10)	Adjuvant Dendritic Cell Immunotherapy complementing standard therapy in High-grade Glioma and Diffuse Intrinsic Pontine Glioma	WT1 mRNA-loaded autologous monocyte derived DC: Phase I newly diagnosed: combined with first line chemoradiation treatment Phase II prior therapy: Dendritic cell vaccination plus optional conventional antiglioma treatment	Feasibility, Safety	University Hospital, Antwerp, Belgium
NCT02465268 ATTAC-II	Phase II Randomized, Blinded, and Placebo-controlled; Autologous LP-mRNA dendritic cell vaccine with chemotherapy	Adult patients (*n*: 175)	Adjuvant CMV RNA-Pulsed Dendritic Cells with Tetanus–Diphtheria Toxoid Vaccine; Newly Diagnosed Glioblastoma with < 3 cm residual tumor following surgery and completed chemoradiation	mRNA DCs encoding the pp65 neoantigen and LAMP (lysosomal associated membrane protein) with GM-CSF vs. placebo and unpulsed PBMC combined with adjuvant TMZ	OS	Immunomic Therapeutics, Inc.; University of Florida; NCI
NCT03688178 DERIVe	Phase II Randomized, Blinded; Autologous LP-mRNA dendritic cell vaccine alone or combined with CD27 mab	Adult patients (*n*: 80)	Adjuvant CMV pp65-LAMP mRNA-pulsed autologous DCs ± Varlilumab; Newly Diagnosed Glioblastoma with < 1 cm residual tumor following surgery and completed chemoradiation	Adjuvant CMV RNA-Pulsed Dendritic Cells with pp65-lysosomal-associated membrane protein DCs ± anti CD27 mAb (Varlilumab) and Td preconditioning during adjuvant TMZ Group 1 and 2 (blinded) Group 3 (nonblinded)	OS Safety Change in Treg Depletion	Duke University Celldex Therapeutics
NCT05357898	Phase I/II first in human, open labelEngineered vaccine alone and combined with chemotherapy	Adult patients (*n*: 60)	Recurrent, locally advanced, or metastatic HPV16+ solid tumors (head and neck, cervical, anal, vulvar, or penile cancer)	SQZ-eAPC-HPV vaccine (mRNA engineered APC-targeting multiple tumor antigens and encoding cytokines) as monotherapy and in combination with pembrolizumab	Safety, Dose-finding	SQZ Biotechnologies
NCT03548571 DEN-STEM	Phase II–III; Open, randomized study mRNA pulsed dendritic cell therapy vs. standard therapy	Adult patients (*n*: 60)	Newly diagnosed IDH wild-type, MGMT-methylated glioblastoma with <1 mm^3^ residual tumor following surgery and completed chemoradiation	Adjuvant autologous trivalent dendritic cells transfected with tm stem cells, survivin, and hTERT combined with TMZ compared to TMZ after surgery and RT	PFS	Oslo University Hospital
NCT04382898 PRO-MERIT	Phase I–II; Open label Dose expansion of W_pro1 vaccine alone and combined with PD-1 inhibitor	Adult patients (*n*: 130)	Metastatic castration-resistant prostate cancer (mCRPC) progressing after 2–3 prior lines of treatment; localized high risk prostate cancer (LPC)	W_pro1 liposomal mRNA vaccine encoding 5 tumor antigens Part 1, Part 2-1B (mCRPC): dose finding; Part 2-1A (mCRPC): vaccine + Cemiplimab Part 2-2 (LPC): vaccine; Part 2-3 (LPC): vaccine + Cemiplimab	Safety, ORR	BionTech SE
NCT03739931	Phase I Open label, dose escalation study of mRNA-2752 alone and combined with PD-L1 inhibition	Adult patients (*n*: 264)	Advanced or metastatic solid tumor malignancies (TNBC, HNSCC, NSCLC, urothelial cancer, melanoma) or lymphoma progressing after standard 1 line of prior therapy	Arm A: mRNA 2752 alone Arm B: mRNA 2752 + Durvalumab	Safety, ORR	ModernaTX, Inc. AstraZeneca
NCT03788083 TMBA	Phase I Open label, intratumoral TriMix injection compared with placebo	Adult patients (*n*: 36)	Newly diagnosed stage 1–2 breast cancer; intratumoral administration before surgery	Dose escalation of TriMix (naked mRNA vaccine encoding CD70, CD40 ligand, and constitutively active TLR4 that activate dendritic cells)	Safety; Immune-modulatory Effect	Universitair Ziekenhuis, Brussels
**Nonvaccine therapies**						
NCT04981691 (Amaretto)	Phase I, mRNA-engineered anti-Mesothelin CAR-T cells therapy	Adult patients (*n*: 12)	Unresectable or metastatic mesothelin expression-positive, advanced solid tumors	Dose-escalation of mRNA transduced mesothelin expressing CAR-T cells	Safety	Ruijin Hospital UTC Therapeutics Inc
NCT04683939	Phase I/IIa dose escalation; Open label; BNT 141 alone and combined with chemotherapy	Adult patients (*n*: 96)	Unresectable or metastatic Claudin 18.2 (CLDN18.2)-positive GI, hepatobiliary or ovarian cancer	Part 1a: Dose-escalation monotherapy with BNT 141 (mRNA-encoded mAb targeting claudin 18.2) Part 1b: Dose escalation with Nab-Pac and gemcitabine	Safety, Dose finding	BionTech SE
NCT04995536	Phase I CpG-STAT3 siRNA combined with RT	Adult patients (*n*: 18)	Recurrent/Refractory B-cell NHL; ≥2 prior lines treatment	Dose escalation of siRNA targeting TLR9 and STAT3 with local RT	Safety, Dose finding	City of Hope Medical Center NCI
NCT05392699	Phase I ABOD2011 hsc IL-12 mRNA	Adult patients (*n*: 60)	Recurrent/Refractory solid tumors progressing after standard therapy	ABOD2011 (Humanized Single chain mRNA encoding IL-12)	Safety, Dose finding	Cancer Institute and Hospital, Chinese Academy of Medical Sciences

Abbreviations: HNSCC: head and neck squamous cell cancer; KRAS: Kirsten rat sarcoma virus; NSCLC: nonsmall cell lung cancer; SCLC: small cell lung cancer; MSI-H: microsatellite instability—high; TMB: tumor mutation burden; AML: acute myeloid leukemia; BRAF: murine sarcoma viral oncogene homolog B; NY-ESO-1: New York esophageal squamous cell carcinoma-1; MAGE-A3: melanoma-associated antigen 3; TPTE: tyrosine-protein phosphatase; MGMT: O-6-methylguanine-DNA methyltransferase; TMZ: temozolamide; RT: radiotherapy; GI: gastrointestinal; Nab-Pac: nao-bound paclitaxel; NHL: non-Hodgkin lymphoma; STAT: signal transduction and activator of transcription 1.

## Data Availability

No new data were created in this study. Data sharing is not applicable.
